# Diffusion of Protease into Meat & Bone Meal for Solubility Improvement and Potential Inactivation of the BSE Prion

**DOI:** 10.1371/journal.pone.0000245

**Published:** 2007-02-28

**Authors:** Brian A. Coll, Rafael A. Garcia, William N. Marmer

**Affiliations:** 1 United States Department of Agriculture (USDA), Agricultural Research Service (ARS), Eastern Regional Research Center, Fats, Oils and Animal Coproducts Research Unit, Wyndmoor, Pennsylvania, United States of America; 2 Widener University, Department of Chemical Engineering, Chester, Pennsylvania, United States of America; Cairo University, Egypt

## Abstract

**Background:**

Government-imposed feed bans have created a need for new applications for meat & bone meal (MBM). Many potential new applications require MBM protein to be both soluble and free of infectious prion. Treatment with protease is generally effective in reducing insoluble, thermally-denatured proteins to soluble peptides. It has been reported in the literature that certain proteases, including Versazyme™, are able to degrade infectious prions in a system where the prions are readily accessible to proteolytic attack. Prions distributed within MBM, however, may conceivably be protected from proteases.

**Methodology/Principal Findings:**

The overall rate of proteolytic MBM digestion depends greatly on whether the protease can penetrate deep within individual particles, or if the protease can only act near the surface of the particle. This research examined the barriers to the diffusion of Versazyme™ into particles of MBM. Confocal microscopy demonstrated differences in the density distributions between the bone and the soft tissue particles of MBM. By tracking the diffusion of fluorescently labeled Versazyme™ through individual particles, it was found that bone particles show full Versazyme™ penetration within 30 minutes, while penetration of soft tissue particles can take up to four hours, depending on the particle's diameter. From the variety of normal proteins comprising MBM, a specific protein was chosen to serve as a prion surrogate based on characteristics including size, solubility, distribution and abundance. This surrogate was used to measure the effect of several factors on Versazyme™ diffusion.

**Conclusions/Significance:**

Results showed that surrogate distributed in bone particles was more susceptible to degradation than that in soft tissue particles. Three factors controllable by unit operations in an industrial-scale process were also tested. It was found that removing the lipid content and hydrating MBM prior to incubation both significantly increased the rate of surrogate degradation. In a test of particle size, the smallest collected diameter range demonstrated the largest degradation of the prion surrogate, suggesting milling would be beneficial.

## Introduction

Meat & bone meal (MBM) is a product from the rendering of the unmarketable animal tissue, primarily the bones and offal from slaughtered livestock, the carcasses of deadstock, and meat products that have exceeded their ‘sell-by’ dates [1]. Prior to the United Kingdom's outbreak of bovine spongiform encephalopathy (BSE) in the 1980s, almost all MBM was utilized as a high-protein ingredient in animal feed. Today, most countries do not allow MBM containing any amount of ruminant tissue to be fed to ruminant animals. In the United States, MBM with ruminant tissue is used in feed for non-ruminant farm animals (especially poultry and swine), companion animals, and aquaculture species, which, with the exception of cats, have never been shown to contract BSE under normal circumstances [2], [3]. In the European Union, MBM is banned from the feed of any animal that may become human food [4]. In the EU, MBM is now primarily either incinerated or used for its energy content in operations such as cement plants [5], [6], or used as an ingredient in pet food. The Canadian government has recently passed a law that will ban certain cattle tissues (known as “specified risk materials”) from all animal feeds, pet foods, and fertilizers [7].

While established outlets for MBM are threatened, the supply of MBM is tied to meat production and thus relatively unresponsive to changes in demand. The development of alternative outlets for MBM is impeded by a couple of important barriers. Most proposed applications for MBM, other than as a fuel, would take advantage of the functional properties of MBM protein. These functional properties are inaccessible unless the highly degraded MBM protein is somehow made soluble, usually by hydrolysis [8]–[11]. An application that successfully harnesses the protein's functional properties could be rejected due to concerns of BSE prion contamination.

BSE prions are relatively resistant to hydrolysis, compared to other proteins [12]. Prion-contaminated tissue can be rendered noninfective by extended alkaline hydrolysis [13]–[15], but the resulting material is extremely degraded and salty and retains little value. Several research groups have identified enzymes capable of digesting prion proteins [16]–[19], while other groups have developed methods to increase the prion's susceptibility to protease digestion [20], [21]. However, all past demonstrations have presented the prions to the proteases in a ‘best case’ scenario; typically raw, homogenized neural tissue diluted with buffer is treaded with the enzyme. These scenarios ignore the mass transport barriers the MBM could impose, limiting access of enzyme to prions distributed within MBM particles. Hypothetically, prions could be protected from enzymatic attack by the matrix of rendered soft tissue or bone in which they would exist. The enzyme may not be able to diffuse into fat-laden particles or calcified bone tissue. Further, the overall rate of proteolytic MBM digestion depends greatly on whether the protease can penetrate deep within individual particles, or if the protease can only act near the surface of the particle. Enzymatic digestion from the surface only might be too slow for practical use.

The present research uses the commercial protease Versazyme™, and treats its ability to inactivate BSE prions as a given, based on previous literature. The factors that affect the ability of this enzyme to penetrate MBM particles are studied. The results provide information critical to the design of a process to simultaneously inactivate MBM prions and add functionality to normal MBM protein.

## Materials and Methods

Meat & bone meal was obtained by the Fats and Proteins Research Foundation (Alexandria, VA) from a member rendering firm and provided to the researchers without revealing the identity of the manufacturer, as described previously [1]. The anonymous manufacturer provided detailed information on its raw material and processing method; this information indicated that the MBM was made entirely from cattle tissue, using a continuous, dry rendering process.

Versazyme™ was purchased from Bioresource International (Morrisville, NC). Before use, Versazyme™ was dissolved in *digestion buffer* (see Table 1) and centrifuged to remove insoluble impurities. All directly compared experiments used Versazyme™ from a single manufacturing lot.

**Table 1:** Reaction and analysis solutions

	Digestion buffer	Mineral Extraction Solution	Protein Extraction Solution	SDS-PAGE loading buffer
pH	8.0	7.4	8.0	8.0
Tris (M)	0.01	-	0.01	0.01
EDTA (M)	-	0.5	-	0.001
Urea (M)	-	-	7.0	-
Thiourea (M)	-	-	2.0	-
Sodium dodecyl sulfate (% w/v)	-	-	2.5	2.5
N-Lauroylsarcosine (% w/v)	-	-	1	-
2-Mercaptoethanol (% w/v)	-	-	-	5.0
Dithiothreitol (M)	-	-	0.05	-
Protease inhibitor cocktail (% v/v)	-	-	0.02	-
Sodium azide (% w/v)	0.05	-	-	-

The fluorescent label used was Alexa Fluor 633 (Molecular Probes, Eugene, OR). All other chemicals used were of reagent or molecular biology grade.

### Fluorescent labeling

Before labeling, solutions of Versazyme™ were fractionated by size exclusion chromatography to remove protein impurities. Using a Biologic DuoFlow Chromatography System (BIO-RAD, Hercules, CA), a Superose 12 10/300 GL column (Tricorn, Uppsala, Sweden) equilibrated with 0.05 M sodium phosphate buffer, 0.15 M NaCl, pH 8.0 at 4°C was calibrated using molecular weight standards. When processing solutions of Versazyme™, eluent fractions predicted to contain Versazyme™ were collected and lyophilized.

The purified enzyme was treated with the fluorescent label according the manufacturer's directions. Briefly, a solution of Alexa Fluor 633 in DMSO was added dropwise to a buffered solution of Versazyme™. After one hour incubation, the protein-label conjugates were separated from unreacted label using a HiTrap Desalting column (Amersham Biosciences, Uppsala, Sweden).

### Confocal microscopy

The imaging system used consisted of an IRBE inverted light microscope, connected to a TCS-SP1 Confocal Scanner Head controlled by LCS-SP2 Leica Confocal Software (all components from Leica Microsystems, Exton, PA). Images of the MBM particles were produced using a 488 nm excitation laser while viewing emission wavelengths from 500 nm to 565 nm. Images showing the distribution of fluorescently-labeled Versazyme™, were produced using a 633 nm excitation laser, while viewing emission wavelengths from 650 nm to 710 nm.

A dilute solution with or without labeled enzyme was mixed with MBM and this suspension was quickly transferred to a viewing dish. Using the continuous scan function, the field of view, magnified to 20 times its true size, was moved until a suitable particle was located, and then the magnification was increased by 4 or 8-fold depending on the size of the particle. The z-plane focus was then adjusted to show the plane passing approximately through the center of the particle. In experiments with enzyme, images were recorded at 0, 10, 20, 30, 40, 50, 60, 90, 120, 150, 180, 240, and 300 minutes. The process was conducted with three bone and three soft tissue particles, which were selected at random.

### Enzymatic digestion

Experiments involving the proteolytic digestion of MBM used a solution of Versazyme™ (0.2 mg/mL in protein solubilization experiments; 0.1 mg/mL in target protein experiments) in *digestion buffer* (see Table 1) at 50°C with constant shaking. MBM was added to this solution at 1% (w/v). Control samples omitted Versazyme™. At the end of a digestion period, reactions were incubated at 90–100°C for 5 minutes to inactivate the enzyme.

### Protein concentration assay

The amount of protein released into solution during an enzymatic digestion was determined by a standard bicinconinic acid protein concentration assay [22], using bovine serum albumin to construct the standard curve.

### Analysis of target protein hydrolysis

After a four-hour digestion, reaction suspensions were centrifuged and the supernatants were discarded, in order to remove soluble protein. The pellets were washed with deionized water and the remaining solids were dried overnight in a vacuum oven at 40°C.

Each sample was then partially defatted by mixing with chloroform for 5 minutes and then pouring through a Büchner funnel fitted with Whatman #50 filter paper. To extract bone mineral that might inhibit target protein solubility, samples were then shaken with 10 mL of *mineral extraction solution* (see Table 1) at 4°C for 3 days. After this treatment the samples were centrifuged and the supernatant was discarded.

After defatting and demineralizing, the target protein was extracted from the solid material by mixing for four hours with 10 mL of a very aggressive protein-solubilizing solution, described in Table 1 (*protein extraction solution*). This suspension was centrifuged at 4°C and the supernatant was collected.

Extract concentration and exchange of the *protein extraction solution* for *SDS-PAGE loading buffer* (Table 1) was achieved using Amicon Diaflow Ultrafiltration Cells (Amicon, Lexington, MA) fitted with 5,000 Da molecular weight cut off Molecular/Por Cellulose Ester ultrafiltration membranes (Spectrum, Rancho Dominguez, CA). These concentrated extracts were analyzed by SDS-PAGE using the Phastsystem (Pharmacia, Uppsala, Sweden). Phastgels with an 8–25% polyacrylamide gradient were used according to the manufacturer's protocol. The gels were stained overnight with SYPRO Ruby Protein Gel stain (Sigma, St. Louis, MO) according to the manufacturer's directions.

To quantitate the target protein band on the gels, they were scanned on a FLA-5000 Fluorescent Image Analyzer (Fujifilm, Tokyo, Japan). The resulting image was analyzed using Multi Gauge v2.02 (Fujifilm, Tokyo, Japan) quantitation software. The concentration of target protein was determined by linear interpolation between the concentration of a standard band of known concentration and a clear background region on the gel.

### Testing different diffusion limiting factors

To prepare partially defatted MBM, 3 g MBM was extracted with 80 mL chloroform and then filtered through a Büchner funnel. To prepare fractions of MBM that were primarily soft tissue or bone particles, a heavy-fluid method adapted from Nash and Mathews [23] was used. Approximately 4 g MBM and 80 mL of chloroform were added to a graduated cylinder and stirred to break apart any multi-particle clumps. This suspension was allowed to settle for 5 minutes, during which a large majority of the soft tissue particles float to the surface and the bone particles sink to the bottom. The liquid and suspended particles were then poured off and filtered, leaving behind the bone particles. To prepare highly hydrated MBM, 100 mg MBM was suspended in 9 mL *digestion buffer* and incubated in a shaking water bath set at 25°C, 175 rpm for 20 hours. To prepare MBM fractions of different size ranges, 10 g MBM was loaded on the top of a stack of half-height sieves (No. 7, 18, 25, 45, 60, 120 - US Alternative sieve designation system), and shaken for 20 minutes on a Ro-Tap Testing Sieve Shaker (W.S. Tyler Company, Cleveland, OH). From this fractions labeled ‘small’ (passing through a sieve with 250 µm openings, but retained on a sieve with 125 µm openings), ‘medium’ (710 µm, 355 µm), and ‘large’ (2800 µm, 1000 µm) were obtained.

## Results

**Figure 1:**
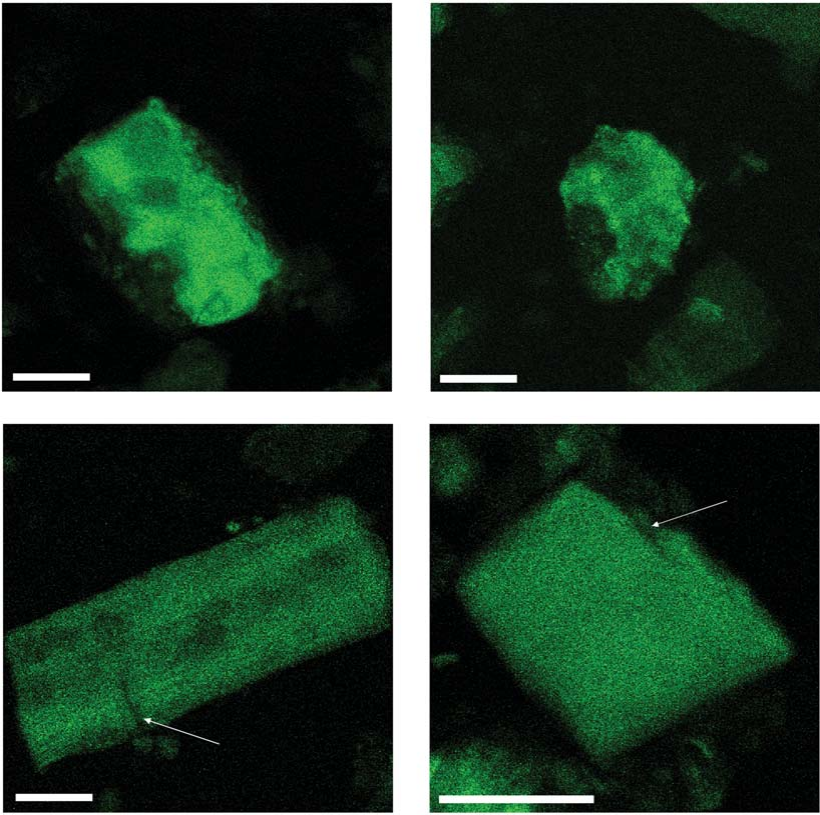
Representative MBM particles autofluorescing. Particles in top row are soft tissue, particles in bottom row are bone. Arrows indicate ‘fissures’; white bar is 25 µm.

Microscopic images of MBM particles were produced using MBM's inherent fluorescence, which is represented by green color in Figure 1. Soft tissue and bone particles were easily differentiated in these images; qualitatively, soft tissue particles were more irregular in shape and more heterogeneous in density, compared to bone particles. Many bone particles had fissures, extending from the surface of the particle inward.

**Figure 2:**
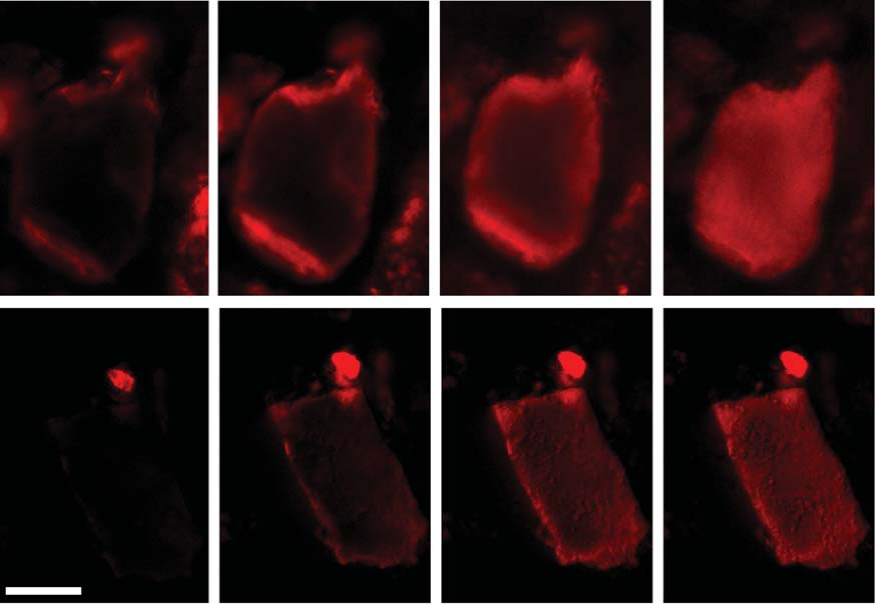
Fluorescently labeled Versazyme™ infusing into MBM particles. Top row (left to right) is a soft tissue particle 0, 20, 90 and 300 minutes after exposure to the Versazyme™ solution. Bottom row is a bone particle 0, 10, 30 and 60 minutes after exposure. White bar is 25 µm.

**Figure 3:**
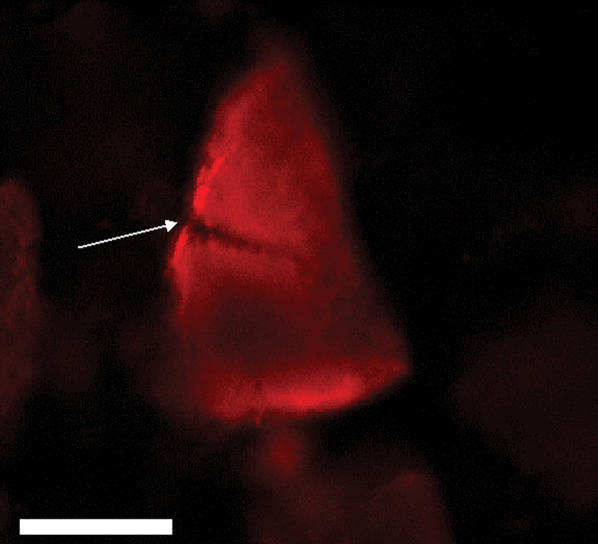
Penetration of fluorescently labeled Versazyme™ (represented by red color) into a bone particle, after 10 minutes incubation. Arrow indicates a fissure in the particle. White bar is 25 µm.

When bathed in a solution of fluorescently labeled Versazyme™, the enzyme was not obviously excluded from any portions of either soft tissue or bone particles, given enough time (Figure 2). The images illustrate the pattern of progressive enzyme infusion observed in all particles studied. Labeled enzyme tended to reach the centers of bone particles long before reaching the center of soft tissue particles. It was apparent that Versazyme™ infused much more freely along fissures in bone particles (Figure 3). Under the conditions tested, Versazyme™ reached the center of particles before they were visibly eroded due to proteolysis.

**Figure 4:**
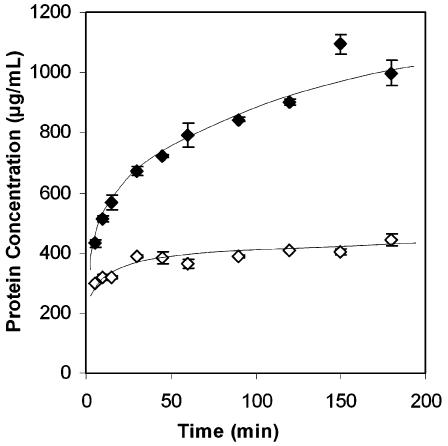
The increase in the solubility of MBM protein as proteolytic digestion progresses. Open symbols represent control experiment with Versazyme™ omitted, closed symbols represent experiment with Versazyme™ (n = 2, each data point; error bars represent ±1 standard deviation).

MBM protein resists dissolution under most conditions, including the conditions used in the present work (Figure 4). Versazyme™ catalyzes the hydrolysis of MBM protein, reducing insoluble MBM proteins to peptides small enough to dissolve into the surrounding solution. Comparison of Figures 2 and 4 suggests that this proteolysis is occurring throughout the particle rather than just at the surface. The Versazyme™ treated samples in Figure 4 release approximately 20% of their total protein into solution in 180 minutes; the images in Figure 2 show that Versazyme™ has penetrated deep into the MBM particles within 180 minutes.

**Figure 5:**
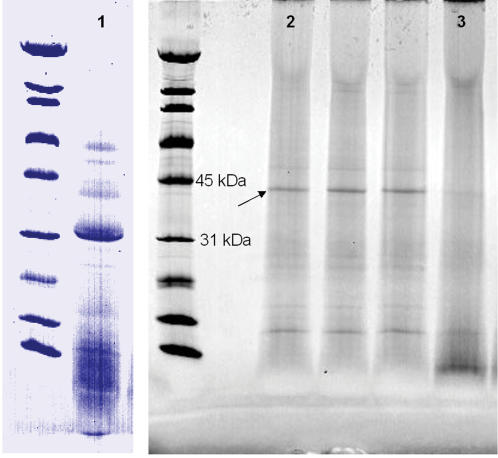
SDS-PAGE. Lane 1 is crude Versazyme™, lane 2 is extract from MBM incubated without Versazyme™, lane 3 is extract from MBM incubated with Versazyme™. Arrow indicates band chosen as prion surrogate.

Quantitative measurements on the relative importance of various diffusion-inhibiting factors were achieved using a prion surrogate. Rather than attempting to spike MBM with a prion surrogate, a protein that occurs naturally in MBM was selected as the surrogate. A 44 kDa protein (Figure 5) was chosen because it met the following criteria: it is insoluble in the conditions used for the enzymatic reaction, it is soluble and can be extracted under special conditions so that it can be quantified by SDS-PAGE and densitometery, it is present in both soft tissue and bone particles at relatively high concentration, it can be hydrolyzed by Versazyme™, and it has a relatively high molecular weight. No attempt was made to determine the identity of this surrogate protein.

**Figure 6:**
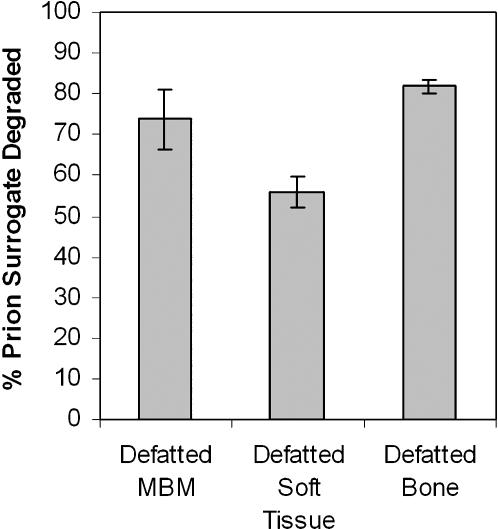
The effect of tissue type on the extent of prion surrogate degradation (n = 3, each data point; error bars represent ±1 standard deviation).

Soft tissue and bone particles differ significantly in the amount of diffusion resistance they present (Figure 6). The method used to fractionate MBM into soft tissue and bone particles involves floatation in chloroform, which has the side effect of partially defatting the particles. To control for the effect of this defatting, surrogate degradation in soft tissue and bone particles is compared to surrogate degradation in MBM which was partially defatted in the same manner. Considerably less surrogate was degraded in the soft tissue particles, indicating that they presented greater obstacles to diffusion.

**Figure 7:**
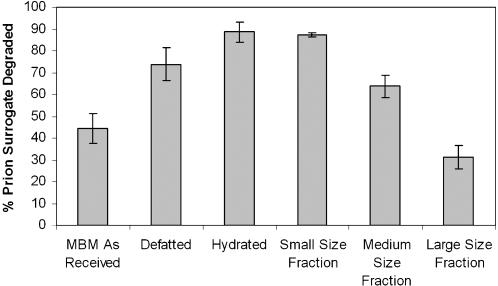
The relative effect of hydration, defatting and particle size on the extent of prion surrogate degradation (n = 3, each data point; error bars represent ±1 standard deviation).

Compared to untreated MBM, MBM that has been defatted, rehydrated, or reduced in size presents less diffusion resistance (Figure 7). Approximately twice as much surrogate was degraded in particles that were either very small or well hydrated, compared to untreated. Partial defatting had a smaller, but still significant effect.

## Discussion

It is reasonable to question whether molecules as large as enzymes can diffuse passively into dense MBM particles. Past studies with plant tissue have often found that without assistance, enzymes penetrate too slowly for practical purposes. Typically, application of pressure or vacuum is required to accelerate infusion; these treatments tend to force enzyme solution into air-filled pores in the tissue. Culver *et al.* [24] found that without vacuum treatment, infusion of α-amylase into apple cubes was too slow to be detected. Pectinase fails to penetrate citrus peels even with pressure or vacuum treatments, unless the outermost layer of the peel is first mechanically scored [25]. Although there has been significant study on the diffusion of small molecules into animal tissue, for purposes such as the curing of ham [26], [27], there are few comparable studies using enzymes. One recent study [28] successfully infused microbial transglutaminase into cattle hides, but without respect to infusion rate.

In the present study, we observed that enzyme can penetrate some portions of an individual MBM particle more rapidly than others (Figures 2 and 3), resulting in a ‘diffusion front’ of varying depth around the perimeter of a particle. Somewhat similar results have been obtained with plant tissue. Varzakas *et al.* [29] observed an irregular pattern of enzyme uptake by soybeans, which they attributed to heterogeneity in cell type and orientation throughout the bean. It is likely that similar factors affect the penetration of Versazyme into MBM particles, but because MBM particles originate in a variety of anatomical locations, cell type and orientation probably varies widely from particle to particle. Differences in tissue structure must account for the differences in diffusion resistance between bone and soft tissue (Figure 6); contrary to our expectations, rendered bone tissue does not provide an enzyme-proof coating for bone protein.

### Conclusion

The treatment of MBM with protease to increase solubility and inactivate prions is technically possible. Neither tissue type presents an insurmountable *physical* barrier to attack by Versazyme™, or presumably, other proteases of similar size. Soft tissue particles' greater resistance to enzyme infusion is concerning, because these particles are more likely than bone particles to have a high prion load.

Enzymatic treatments are often dismissed as being prohibitively expensive for price-sensitive applications. This argument is becoming progressively less valid as the enzyme-producing industry matures, and enzyme prices drop. The enzyme used this research is marketed primarily as an additive for poultry rations.

The practicality of a protease-MBM treatment process depends largely on whether it can be designed to work rapidly with a relatively small amount of enzyme. This research shows that additional unit operations such as milling, solvent extraction, and hydration improve the performance of the enzyme in such a process by allowing the enzyme to rapidly penetrate and hydrolyze throughout the particle, rather than just acting on the particle's surface. Further improvements might be achieved by adopting the pressure or vacuum treatments that have been used to force enzyme solution into plant tissue, but this depends largely on the existence of gas-filled spaces within the particles.
